# Dr. George W. Grabenstatter

**Published:** 1917-02

**Authors:** 


**Dr. George W. Grabenstatter,** Buffalo 1900, of Buffalo, died at the General Hospital, Jan. 3, after a second operation for appendicitis. He was born in Buffalo in 1871. After graduation, he served at the Fitch Hospital as interne and then entered the U. S. Army, serving as surgeon in the Philippines for two years, then spending a year in travel through China and other eastern countries. He had practiced in Buffalo since 1903.

